# HIV, violence, blame and shame: pathways of risk to internalized HIV stigma among South African adolescents living with HIV

**DOI:** 10.7448/IAS.20.1.21771

**Published:** 2017-08-21

**Authors:** Marija Pantelic, Mark Boyes, Lucie Cluver, Franziska Meinck

**Affiliations:** ^a^ Department of Social Policy and Intervention, Oxford University, Oxford, UK; ^b^ International HIV/AIDS Alliance, Brighton, UK; ^c^ School of Psychology and Speech Pathology, Curtin University, Perth, Australia; ^d^ Department of Psychiatry and Mental Health, University of Cape Town, Cape Town, South Africa; ^e^ OPTENTIA, School of Behavioural Sciences, North West University, Vanderbeijlpark, South Africa

**Keywords:** adolescent, stigma, abuse, shame, HIV/AIDS, structural equation modelling

## Abstract

**Introduction**: Internalized HIV stigma is a key risk factor for negative outcomes amongst adolescents living with HIV (ALHIV), including non-adherence to anti-retroviral treatment, loss-to-follow-up and morbidity. This study tested a theoretical model of multi-level risk pathways to internalized HIV stigma among South African ALHIV.

**Methods**: From 2013 to 2015, a survey using **t**otal population sampling of ALHIV who had ever initiated anti-retroviral treatment (ART) in 53 public health facilities in the Eastern Cape, South Africa was conducted. Community-tracing ensured inclusion of ALHIV who were defaulting from ART or lost to follow-up. 90.1% of eligible ALHIV were interviewed (*n* = 1060, 55% female, mean age = 13.8, 21% living in rural locations). HIV stigma mechanisms (internalized, enacted, and anticipated), HIV-related disability, violence victimization (physical, emotional, sexual abuse, bullying victimization) were assessed using well-validated self-report measures. Structural equation modelling was used to test a theoretically informed model of risk pathways from HIV-related disability to internalized HIV stigma. The model controlled for age, gender and urban/rural address.

**Results**: Prevalence of internalized HIV stigma was 26.5%. As hypothesized, significant associations between internalized stigma and anticipated stigma, as well as depression were obtained. Unexpectedly, HIV-related disability, victimization, and enacted stigma were not directly associated with internalized stigma. Instead significant pathways were identified via anticipated HIV stigma and depression. The model fitted the data well (RMSEA = .023; CFI = .94; TLI = .95; WRMR = 1.070).

**Conclusions**: These findings highlight the complicated nature of internalized HIV stigma. Whilst it is seemingly a psychological process, indirect pathways suggest multi-level mechanisms leading to internalized HIV stigma. Findings suggest that protection from violence within homes, communities and schools may interrupt risk pathways from HIV-related health problems to psychological distress and internalized HIV stigma. This highlights the potential for interventions that do not explicitly target adolescents living with HIV but are sensitive to their needs.

## Introduction

There are an estimated 2.1 million adolescents living with HIV (ALHIV) worldwide and 85% of them live in Sub-Saharan Africa [1]. South Africa is home to the world’s largest population of ALHIV [2]. Globally, AIDS-related mortality is on the rise among ALHIV: between 2005 and 2015, AIDS-related mortality more than doubled in this high-risk population whilst all other age groups experienced reductions [3].

Internalized HIV stigma occurs when a person living with HIV internalizes perceived negative public attitudes towards people living with HIV and accepts them as applicable to themself [4]. By evoking strong feelings of shame and worthlessness [4], internalized HIV stigma can pose a serious threat to the long-term survival of ALHIV [5–7]. There are no quantitative studies on the prevalence or drivers of internalized HIV stigma among ALHIV in Sub-Saharan Africa [4]. In order to identify points for intervention for these adolescents, it is essential to understand mechanisms of risk for internalized HIV stigma in this population.

According to the HIV-stigma framework, internalized stigma is one of three core psychosocial HIV-stigma mechanisms experienced by people living with HIV [8,9]. Enacted HIV stigma refers to the extent to which people living with HIV are discriminated against or treated differently based on their HIV status [10]. Anticipated HIV stigma refers to the extent to which people living with HIV anticipate negative public attitudes or differential treatment related to their HIV status [8]. The few Sub-Saharan African studies that tested associations between internalized HIV-stigma and other stigma mechanisms did this with adult samples, using simple bivariate correlations to assess validity of multidimensional HIV-stigma scales [4]. They found weak-to-moderate associations between enacted and internalized HIV stigma [10–12], and anticipated and internalized HIV stigma [13]. But these analyses were intended for psychometric scale assessments and hence did not take into account any potential confounding pathways or variables.

There is also a dearth of research on other potential drivers of internalized HIV stigma in Sub-Saharan Africa [4] – perhaps a reason for the lack of well-established programmes to address it [14]. A recent systematic review found that poor HIV-related health appeared to be a risk factor in three longitudinal studies, and depression appeared to be a risk factor in two longitudinal studies [4]. No other consistent predictors were found, and no quantitative studies assessing internalized HIV stigma among children or adolescents in the region were identified.

The literature on violence victimization and developmental psychology suggests that child maltreatment may also be a key driver of internalized HIV stigma via reduced psychological wellbeing [15,16]. Longitudinal findings from multiple systematic reviews consistently suggest that over time, child abuse victimization in the form of physical, sexual, emotional abuse and bullying leads to increases in depressive symptoms [17–22]. Similarly, enacted HIV stigma and bullying have been shown to have enduring negative impacts on depressive symptoms among youth from families affected by AIDS, which includes both ALHIV and HIV-negative adolescents [16].

Additionally, it is essential to assess whether and how HIV-related health is associated with maltreatment of ALHIV and internalized HIV stigma. Evidence from South Africa suggests that children from AIDS-affected families are at elevated risk of various forms of maltreatment when compared to their peers [16,23]. The maltreatment is not always exhibited as HIV-specific enacted stigma, but is nonetheless often indirectly tied to the children’s health and HIV status. For example, ethnographic data from Brazil, South Africa, Zambia and Zimbabwe suggest that child abuse of AIDS-affected youth was linked to other household members’ fear of HIV infection, and the perceived added burden of caring for ill children within a context of poverty [24–28]. AIDS-affected youths’ experiences of abuse victimization were thus inextricably tied to their HIV-related health, and their perceptions of HIV stigma further shaped by abuse victimization. Whilst longitudinal research with adults has established a link between poor HIV-related health and internalized HIV stigma [4], qualitative research with ALHIV suggests that enacted HIV stigma and abuse victimization might account for this relationship [24–28]. However, these hypothesized pathways have not yet been tested quantitatively.

Taken together, this growing body of longitudinal research suggests likely pathways from HIV-related disability to internalized HIV stigma both directly [4], and via social and psychological risks [4,16,23]. Namely, HIV-related disability may place ALHIV at risk of more internalized HIV stigma via increased likelihood of violence victimization and enacted HIV stigma (social risks), and via more anticipated HIV stigma and depression (psychological risks). However, neither of these potential pathways have been tested in structural equation models, nor explored among ALHIV.

**Figure 1.**
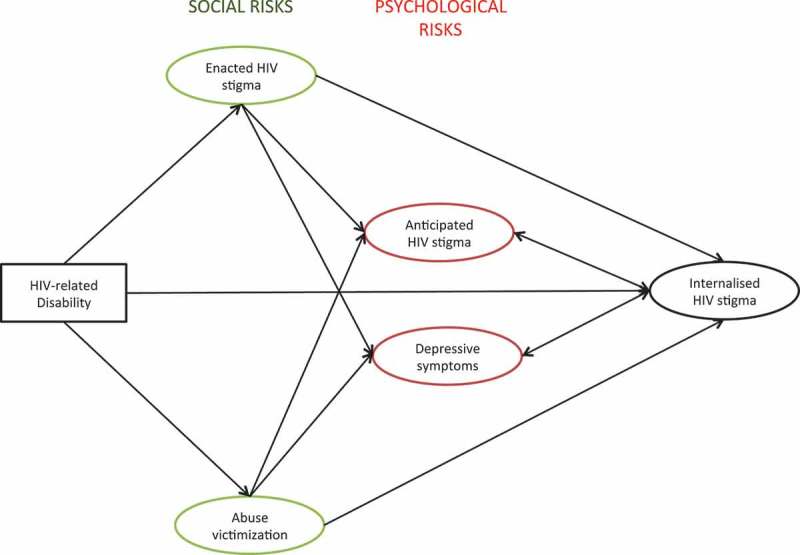
Hypothesized risk pathways from HIV-related disability to internalized HIV stigma.

This study aimed to develop and test a theoretical model of associations between HIV-related disability, hypothesized social (violence victimization and enacted HIV stigma) and psychological (anticipated HIV stigma and depression) risk factors and internalized HIV stigma. It draws on longitudinal quantitative findings from studies with adults living with HIV [4], qualitative research with AIDS affected adolescents in HIV-endemic contexts [24–28] and longitudinal research on psychological impacts of child maltreatment [17–21] to hypothesize pathways of risk (Figure 1).

## Methods

From 2013 to 2015, a survey of ALHIV was administered in the Eastern Cape, South Africa. First, all state healthcare facilities in two health districts were mapped. Facilities providing treatment for 5 or more ALHIV were included in the study (*n* = 53). Second, all adolescents (ages 10–19) who had ever initiated ART in selected health facilities were sampled using clinic files. Patients were eligible if they met the 10–19 age bracket at the time of data collection, and if they were ever initiated on ART that was not short-term post-exposure prophylaxis. Second, adolescents (*n* = 1,176) were traced in their communities so as to ensure inclusion of ALHIV who were not actively engaged in the healthcare system. One-on-one interviews (*n* = 1060) were conducted in participants’ homes, clinics or other places identified as safe and comfortable by the participants.

The questionnaire was pre-piloted with ALHIV, and a systematic review was conducted to ensure inclusion of key variables related to internalized HIV stigma into the questionnaire [4]. The questionnaire was developed in English, and items were translated and back translated independently by different Xhosa and English-speaking research assistants. Interviews lasted for about 90 min and were carried out on tablets. The full questionnaire consisted of nearly 450 items but tablet-assisted data collection allowed for skip patterns that reduced participant burden by omitting irrelevant questions. Participants chose whether they wanted to complete the interview directly on a mobile device (low-cost tablet), with the help of a research assistant, or a mix of the two depending on the questions. In case of low literacy levels or cognitive delay, Xhosa, English and Afrikaans-speaking interviewers, trained in working with ALHIV, read questions and provided assistance. Each completed questionnaire was assigned a serial number, which was recorded in the study roster. Data were linked to the serial number of their questionnaire and not participants’ names resulting in an anonymized dataset. To ensure data protection from potential confidentiality breaches, data were accessible via password known only to the study management team.

Remuneration was not provided except for certificates, refreshments, and a toiletry pack. Voluntary informed consent was obtained from caregivers and adolescents. Where participants reported recent abuse, rape, suicidal attempt or other risk of significant harm, referrals were made to child protection and health services. Ethical clearance for this study was provided by the University of Oxford, University of Cape Town, South African National Departments of Health, Basic Education and Social Development as well as the Eastern Cape Departments of Health, Basic Education and Social Development.

## Measures

Age, gender and rural household location were recorded for descriptive purposes and for inclusion in the model as covariates.

*HIV-stigma mechanisms* were measured via the 10-item HIV-stigma scale for ALHIV (ALHIV-SS). The ALHIV-SS was developed in collaboration with ALHIV in South Africa and has been shown to have strong psychometric properties [29]. Enacted, anticipated and internalized HIV stigma were assessed via 3, 2 and 5 items, with Cronbach’s α levels of .57, .70 and .75 respectively. Latent variables of enacted, anticipated and internalized HIV stigma were used with individual items loaded onto them.

*HIV-related disability* was measured via two items asking about physical and cognitive disability adapted from The International Classification of Functioning, Disability and Health. The scale displayed good reliability (Chronbach’s α = .60) [30]. Items were recoded into a dichotomous variable to capture any report of disability (0: no disability; 1: 1 or more disability).

*Violence victimization* was a latent measure with loaded factor scores for physical, emotional and sexual abuse, and bullying victimization. *Physical abuse* (2 items) and *emotional abuse* (10 items) were measured using items from the UNICEF Measures for National-level Monitoring of Orphans and Other Vulnerable Children (Snider & Dawes, 2006). These measures have displayed strong reliability (α = .70) in previous studies of HIV/AIDS affected adolescents in South Africa [31]. Response options for all physical abuse and emotional abuse items were offered on a 5-point scale (0: never; 1: has happened, but not in the last year; 2: at least once this year; 3: monthly; 4: weekly). *Contact sexual abuse* was measured using three items from the Juvenile Victimization Questionnaire (JVQ) [32], also used in previous studies in South Africa [31]. *Bullying victimization* was measured with the 9-item ‘Social and Health Assessment Peer Victimization Scale’, used in previous studies with AIDS-affected children [16,33,34]. This scale was adapted from the Multidimensional Peer Victimization Scale, which was validated in the US [35]. Items include; being called names, being hit or threatened and having possessions broken or stolen. It demonstrated good internal consistency (α = .79) in the present sample.

*Depressive symptoms* were measured via the Child Depression Inventory short form (CDI-S), which has comparable results with the full CDI [36]. CDI-S has been used with AIDS-affected adolescents in South Africa, displaying acceptable internal consistency (α = .67-.69) [37]. CDI-S also demonstrated acceptable internal consistency in the present sample (α = .62). A latent variable was used in the present study, with individual items loading onto the depressive symptoms factor.

## Analysis strategy

The analysis was conducted in three stages using MPlus. First, to estimate the extent of bias in the sample, socio-demographic characteristics of eligible ALHIV who were not reached were compared to the socio-demographic characteristics of ALHIV who were interviewed. These socio-demographic data were recorded from clinic files. Mean age between the included and excluded ALHIV was compared using z-scores. Gender and rurality frequencies between the included and excluded ALHIV were compared using *Chi*^2^.

Second, prevalence rates of enacted, anticipated and internalized HIV stigma, HIV-related disability, and different types of violence victimization were calculated, and desegregated by gender. Dichotomous variables for different stigma mechanisms were defined to capture reports of any enacted, anticipated and internalized HIV stigma. Similarly, a dichotomous variable for any type of HIV-related disability was calculated. A dichotomous variable was created for *frequent emotional abuse* (0: once this year or less; 1: monthly or more often) and similarly for *frequent physical abuse* (0: once this year or less; 1: monthly or more often). Categorisations of frequent physical and emotional abuse were chosen based on previous studies with vulnerable children in South Africa, which aimed to clearly distinguish abuse from harsh parenting [38]. *Lifetime prevalence of contact sexual abuse* was also assessed (0: never sexually abused; 1: sexually abused). A dichotomous variable for bullying victimization above the mean was calculated. Mean scores for depressive symptoms were also calculated due to a lack of a validated clinical cutoff for the South African context.

Third, the structural equation model consisted of [1] a confirmatory factor analysis to confirm latent constructs and [2] a pathway model to assess a hypothesized theoretical model of internalized HIV-stigma risks (Figure 1). All included variables were latent constructs except for child disability, which was an observed variable. The model controlled for age, gender and rural household location. Model fit was assessed with: Comparative Fit Index (CFI) (>.90 indicates adequate fit), Tucker Lewis Index (TLI) (> .95 indicates adequate/good fit) [39–41], and Root Mean Square Error of Approximation (RMSEA) (< .05 indicated good model fit) [42]. *Chi*^2^ was noted but not used to estimate model fit because it is sensitive to sample size and is prone to Type 2 error [43,44].

## Results

90.1% (*n* = 1060) of the eligible ALHIV were interviewed. 4.1% of ALHIV refused to participate (either caregiver or adolescent), 3.7% could not be traced, 0.9% were excluded due to severe cognitive delays and 1.2% were excluded due to other reasons such as emergency referrals, unsafe communities, and having moved out of study catchment area. The mean age of respondents was 13.8; 55.2% (587) of them were girls and 21.4% (228) lived in rural locations. A comparison of the included and excluded eligible samples based on known information: age, gender and rural/urban residential location identified no statistically significant differences between the interviewed and excluded samples (Table 1).

**Table 1.** Comparisons between included and excluded ALHIV

	HIV+ (*n* = 1060)	Excluded (*n* = 116)	Comparison tests
Age (mean, SD)	13.8, 2.834	14.8, 2.91	*p* = .671
Female (*n*, %)	587, 55.2%	66, 56.9%	*p* = .769
Rural (*n*, %)	228, 21.4%	26, 22.4%	*p* = .813

*p*-Values associated with z score and *chi^2^* tests.

Table 2 provides further sample characteristics on the variables of interest, disaggregated by gender. Girls were more likely than boys to report any anticipated HIV stigma, frequent emotional abuse victimization and lifetime prevalence of sexual abuse victimization. No other gender differences were observed.

**Table 2.** Sample characteristics

	Boys living with HIV (*n* = 476)	Girls living with HIV (*n* = 584)	All ALHIV (*n* = 1060)	Gender comparison tests* (*p* value)
HIV-related disability	195, 41.0	238, 40.8	433, 40.8	.944
Internalized HIV stigma	103, 22.0	140, 25.3	243, 22.9	.206
Anticipated HIV stigma	116, 24.4	199, 34.1	315, 29.7	.001
Depression (mean, SD)	1.2, 1.8	1.3, 2.1	1.3, 2.0	.779
Enacted HIV stigma	31, 6.5	46, 7.9	77, 7.2	.400
Frequent physical abuse	28, 5.9	21, 3.6	49, 4.6	.078
Frequent emotional abuse	27, 5.7	52, 8.9	79, 7.5	.046
Lifetime prevalence of sexual abuse	17, 3.6	48, 8.2	65, 6.1	.002
Bullying victimization	162, 34.0	178, 30.5	340, 32.1	.218

*Chi^2^ and *t tests* were used to examine gender differences for dichotomous variables and scale variables, respectively.

### Measurement model

The measurement model confirmed distinct latent constructs for enacted HIV stigma, violence victimization, anticipated HIV stigma, depression and internalized HIV stigma. Table 3 summarizes factor loadings of each indicator onto latent constructs of abuse victimization, enacted HIV stigma, anticipated HIV stigma, depression and internalized HIV stigma. The measurement model statistics indicated excellent model fit: RMSEA = .04; CFI = .984; TLI = .982; WRMR = .873; Chi^2^(df) = 297.935 (242).

**Table 3.** Factor loadings for latent constructs

	Standardized estimate
**Abuse victimization**	
Physical abuse	.458***
Emotional abuse	.659***
Sexual abuse	.407***
Bullying victimization	.648***
**Enacted HIVstigma**	
Stopped spending time with friends	.769***
Lost friends because of HIV	.674***
Teased because of HIV status	.990***
**Anticipated HIVstigma**	
People think that HIV-positive people are disgusting	.994***
People think that HIV is a punishment	.729***
**Depression**	
Personal outlook	.550***
Frequency of sadness	.695***
Feelings about appearance	.599***
Feelings towards self	.768***
Frequency of loneliness	.529***
Self-evaluation	.456***
Friends	.387***
Frequency of crying	.662***
Feelings of love	.623***
Bothered frequency	.769***
**Internalized HIVstigma**	
Does not feel as good as others because of their HIV status	.766***
Would rather die than live with HIV	.817***
Feels like a bad person for living with HIV	.833***
Feels ashamed of their HIV status	.780***
Feels dirty/contaminated inside because of HIV	.852***
*** indicates *p *< .001	

### Structural model

**Figure 2.**
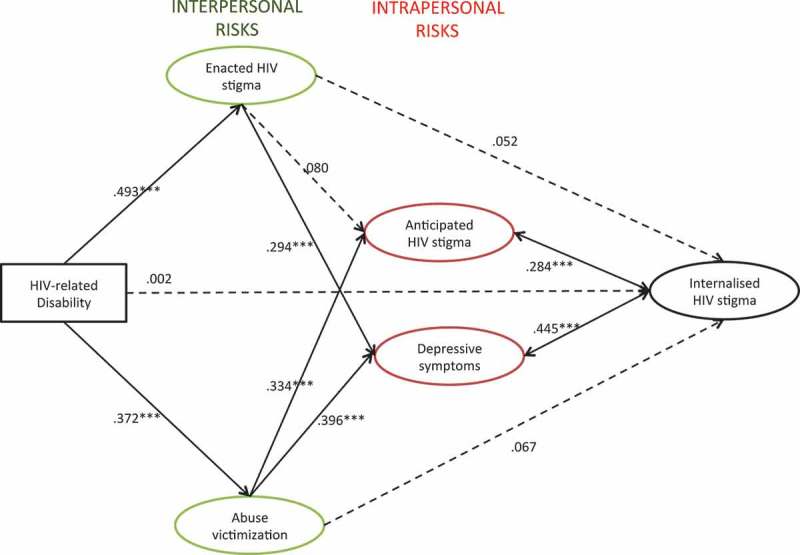
Final structural equation model results. Rectangular shape signifies an observed variable whereas ovals mark latent variables. Values indicate standardized *β* weights. Dotted lines indicate hypothesized pathways that were non-significant. Full lines indicate pathways that were significant. *** indicates *p *< .001; ** indicates *p *< .005; * indicates *p *< .05. Model fit: RMSEA: .023; CFI: .94; TLI: .95; WRMR: 1.070. Model controlled for age, gender, rural household location.

The results of the final structural equation model are summarized in Figure 2. The model controlled for age, gender and rural household location. Rectangular shapes signify observed variables whereas ovals mark latent variables. Values indicate standardized *β* weights. Full lines indicate pathways that were significant at the *p *< .001 level (***). Dotted lines indicate hypothesized pathways that were non-significant. HIV-related disability was hypothesized to have a direct effect on enacted HIV stigma, abuse victimization and internalized HIV stigma. HIV-related disability was hypothesized to predict enacted HIV stigma, abuse victimization and internalized HIV stigma. Enacted HIV stigma and abuse victimization were both hypothesized to be associated with depression, anticipated HIV stigma and internalized HIV stigma. Bidirectional associations between internalized HIV stigma and anticipated HIV stigma and depression were also hypothesized. Of these hypothesized associations, four were non-significant: (1) the direct association between HIV-related disability and internalized HIV stigma, (2) the direct association between abuse victimization and internalized HIV stigma, (3) the direct association between enacted HIV stigma and internalized HIV stigma and (4) the association between enacted HIV stigma and anticipated HIV stigma.

Internalized HIV stigma was directly associated with anticipated HIV stigma (*β *= .284, *p *< .001), depressive symptoms (*β *= .445, *p *< .001) and urban household location (*β *= -.014, *p *< .001). Gender (*β *= .017, *p *= .196) and age (*β *= -.011, *p *= .583) were not significantly associated with internalized HIV stigma. In addition, the following pathways were identified. HIV-related disability was associated with more enacted HIV stigma (*β *= .493, *p *< .001) and more abuse victimization (*β *= .372, *p *< .001). Enacted HIV stigma was associated with more depressive symptoms (*β *= .294, *p *< .001) but not with anticipated HIV stigma. Abuse victimization was associated with more anticipated HIV stigma (*β *= .334, *p *< .001) and more depressive symptoms (*β *= .396, *p *< .001). Indirect effects of HIV-related disability on higher depression scores via more abuse victimization (*β *= .147, *p *< .001) and more enacted HIV stigma (*β *= .145, *p *< .001) were observed. HIV-related disability was also indirectly associated with more anticipated HIV stigma via more abuse victimization (*β *= .124, *p *< .001).

The fit of the final model was RMSEA = .023; CFI = .94; TLI = .95; WRMR = 1.070; Chi^2^ (df) = 3857.655 (372). All fit statistics were excellent according to the pre-specified criteria.

## Discussion

This study aimed to test a theoretical model of hypothesized risk pathways to internalized HIV stigma among ALHIV in South Africa. As hypothesized, significant associations between internalized stigma and anticipated stigma, as well as depression were obtained. Unexpectedly, HIV-related disability, victimization, and enacted stigma were not directly associated with internalized stigma. These findings highlight the complicated nature of internalized HIV stigma. Whilst it is commonly seen as a purely psychological process, indirect pathways suggest multi-level mechanisms leading to internalized HIV stigma.

The present study expands on findings from longitudinal studies with adults living with HIV in the region, which found poor HIV-related physical health to be predictive of increases in internalized HIV stigma [4]. However, in the present sample of ALHIV, HIV-related disability was associated with internalized HIV stigma only indirectly, via social risks (enacted HIV stigma and abuse victimization) and psychological risks (anticipated HIV stigma and depressive symptoms).

A growing corpus of research suggests that internalized HIV stigma hampers uptake of evidence-based HIV prevention, treatment and care [45–49] but most anti-stigma interventions target enacted HIV stigma [14]. There are only three published evaluations of interventions that target internalized HIV stigma in sub-Saharan Africa and these were delivered through clinics to adults living with HIV who were actively engaged in health care [50–52]. In light of present findings, a community-based approach to addressing internalized HIV stigma may be required for adolescents (Table 4). Our findings suggest that addressing individual-level risks – such as anticipated HIV stigma, depression and HIV-related health – will be essential to reduce internalized HIV stigma among ALHIV. However, present findings also suggest that tackling discrimination against ALHIV and violence victimization in homes, schools and communities may be essential to interrupt pathways of risk to internalized HIV stigma.

**Table 4.** Recommendations for policy and practice

**Policy makers**
Policies that do not specifically target ALHIV, but are sensitive to their needs might be beneficial. For example, evidence-based interventions that aim to reduce violence in schools, homes and communities may be beneficial for ALHIV even if they are not specifically designed for this population. Evidence-based policies to support ALHIV and disabilities are urgently needed.
**Healthcare providers**
Taking into consideration the home and school environments of adolescent patients might help better support them. Integrating psychological support and counselling into adolescent HIV care might help adolescents cope with HIV stigma. In resource-limited contexts where psychosocial support is not available for all patients, it may be helpful to prioritize adolescents with physical or cognitive disabilities as they are at heightened risk of abuse and bullying victimization.
**Community organizations**
Family, school and community-based violence prevention programmes might help combat internalized HIV-related shame. Healthcare providers may need support to better address the holistic needs of adolescent patients. Community organizations may help train healthcare providers to (a) routinely screen adolescent patients for mental health difficulties and history of violence victimization and (b) refer young people to adequate support services. Teachers should also be supported and trained to detect bullying, discrimination and violence in schools, and refer adolescent students to adequate psychosocial support services.
**Schools**
Mechanisms to inform teachers of bullying and discrimination in schools are needed. Violence prevention in schools that does not single out students living with HIV but rather targets more broadly peer and teacher violence may be beneficial for ALHIV.

For example, interventions that do not necessarily target HIV-positive adolescents but are sensitive to their needs such as school-based anti-bullying programmes [53] might help reduce internalized HIV stigma. Parenting interventions specifically adapted for resource-limited settings might also help against internalized HIV stigma [54–56]. Youth violence exposure in one environment may impact involvement and/or victimization within another [57]. Therefore, violence prevention programmes simultaneously targeting adolescents’ local communities, homes and schools might be particularly beneficial.

Of note is that enacted HIV stigma was *not* associated with anticipated HIV stigma, which was one of the determinants of internalized HIV stigma. This is contrary to earlier studies, which focused on adults and used simple bivariate correlations [4]. Our findings suggest that HIV-related disability and/or violence victimization predict anticipated HIV stigma over and above enacted HIV stigma. Moreover, our findings suggest that enacted HIV stigma is associated with internalized HIV stigma only indirectly, via higher levels of depressive symptoms. More foundational and intervention research on the various HIV-stigma mechanisms is needed.

Gender differences were not observed for internalized or enacted HIV stigma in the initial descriptive analyses, and gender did not significantly contribute to the final model. Previous research in the region with adult samples generated inconsistent findings on the relationship between gender and internalized HIV stigma. For example, whilst two studies in Uganda and South Africa found that women were more likely to report internalized HIV stigma [58,59], another South African study found that women were less likely than men to report internalized HIV stigma [12]. To our knowledge, this is the first study in the region to look at this association within an adolescent, community-traced sample. Whilst findings challenge the common perception that girls are more likely to experience enacted HIV-related stigma [60–62], we also found that they were more likely to *anticipate* HIV stigma. More research on how gender is associated with different HIV-stigma mechanisms is clearly needed.

This study has six important limitations that should be noted. First, the cross-sectional data limit inferences about directionality. More foundational, longitudinal research is needed to understand the mechanisms and their temporal sequencing. Second, all measures relied on self-report. Third, stigma is culturally embedded and socially constructed [63,64], and therefore HIV-stigma mechanisms might develop differently depending on the context. The present study reports on data from ALHIV in the Eastern Cape, South Africa and this may limit generalizability of findings. Fourth, this study did not examine protective factors. Evidence is urgently needed to better understand whether and how protective factors such as social support [4] may moderate pathways of risk to internalized HIV stigma. Fifth, only adolescents who had ever initiated ART were sampled. This may have limited reach of the most stigmatized adolescents who have not yet tested or initiated ART. However, unlike previous studies on internalized HIV stigma [4], this study community-traced adolescents in an attempt to reach most at-risk adolescents who had dropped out of treatment. Hence, adolescents who were missing clinic visits, defaulting or were lost to follow up were included in the study. Sixth, the length of interviews could have compromised data quality but inclusion of breaks and games sought to minimize participant burden.

This study also has a number of strengths. First, to our knowledge, this is the first study on internalized HIV stigma among ALHIV in Africa. Second, the structural equation model allowed the assessment of multiple inter-related risks. This enabled a more nuanced picture of how HIV-related disability – a key risk factor that had already been detected in previous research [4] – leads to internalized HIV stigma. Fourth, previous, adult-focused research on internalized HIV stigma in the region has largely overlooked interpersonal risk factors [4]. A key contribution of this study is that it empirically highlights that internalized HIV stigma is likely to be shaped by social contexts and power inequalities [65].

## Notes

To access the supplementary material to this article please see Supplementary Files under Article Tools online.
